# Coupling genome-wide continuous perturbation with biosensor screening reveals the potential targets in yeast isopentanol synthesis network

**DOI:** 10.1016/j.synbio.2024.12.010

**Published:** 2024-12-30

**Authors:** Qi Xiao, Jingjing Shi, Lixian Wang, Guoping Zhao, Yanfei Zhang

**Affiliations:** a Tianjin Institute of Industrial Biotechnology, Chinese Academy of Sciences, Tianjin, 300308, China; b National Center of Technology Innovation for Synthetic Biology, Tianjin, 300308, China; c College of Life Sciences, University of Chinese Academy of Sciences, Beijing, 101408, China; d CAS-Key Laboratory of Synthetic Biology, CAS Center for Excellence in Molecular Plant Sciences, Institute of Plant Physiology and Ecology, Chinese Academy of Sciences, Shanghai, 200032, China

**Keywords:** Isopentanol production, Genomic perturbation, Biosensors, Transcriptome analysis

## Abstract

The increasing consumption of fossil fuels is contributing to global resource depletion and environmental pollution. Branched-chain higher alcohols, such as isopentanol and isobutanol, have attracted significant attention as next-generation biofuels. Biofuel production through microbial fermentation offers a green, sustainable, and renewable alternative to chemical synthesis. While enhanced production of isopentanol has been achieved in a variety of chassis, the fermentation yield has not yet reached levels suitable for industrial-scale production. In this study, we employed a continuous perturbation tool to construct a genome-scale perturbation library, combined with an isopentanol biosensor to screen for high-yielding mutants. We identified five high-yielding mutants, each exhibiting an increased glucose conversion rate and isopentanol titer. The F2 strain, in particular, achieved an isopentanol titer of 1.57 ± 0.014 g/L and a yield of 14.04 ± 0.251 mg/g glucose (10% glucose), surpassing the highest values reported to date in engineered *Saccharomyces cerevisiae*. Systematic transcriptome analysis of the isopentanol synthesis, glycolysis, glycerol metabolism, and ethanol synthesis pathways revealed that *MPC*, *OAC1*, *BAT2*, *GUT2*, *PDC6*, and *ALD4* are linked to efficient isopentanol production. Further analysis of differentially expressed genes (DEGs) identified 17 and 12 co-expressed DEGs (co-DEGs) in all mutants and the two second-round mutants, respectively. In addition, we validated the knockout or overexpression of key co-DEGs. Our results confirmed the critical roles of *HOM3* and *DIP5* in isopentanol production, along with genes associated with the aerobic respiratory chain (*SDH3*, *CYT1*, *COX7*, *ROX1*, and *ATG41*) and cofactor balance (*BNA2* and *NDE1*). Additionally, functional analysis of the co-DEGs revealed that *MAL33* is associated with the synthesis of branched-chain higher alcohols, expanding the intracellular metabolic network and offering new possibilities for green, cost-effective biofuel production.

## Graphical abstract

**Figure:**
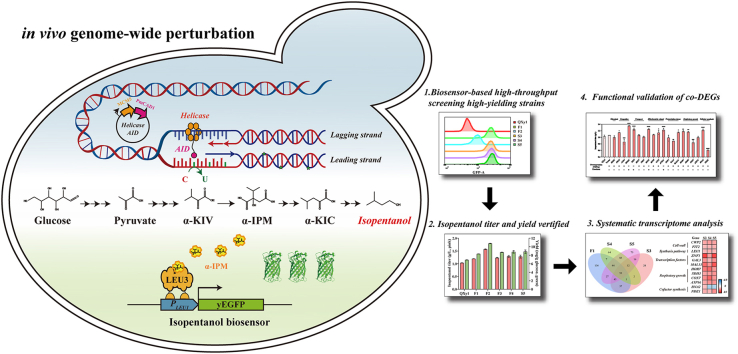

## 1 Introduction

Energy and environmental sustainability are among the most pressing global challenges. Fossil fuels, the primary energy source, are non-renewable, and their combustion produces significant amounts of exhaust gases that contribute to global warming and rising sea levels, both of which are critical climate issues. In contrast, biofuels present a promising alternative to fossil fuels, offering sustainability and renewability [1,2]. Branched-chain higher alcohols, such as isobutanol and isopentanol, exhibit properties closer to petrol than ethanol and are considered the next generation of biofuels [3,4]. Notably, isopentanol can be catalytically converted into jet fuel, providing renewable energy for aviation and serving as a key chemical precursor for industrially important compounds such as isoamyl acetate and polyethylene terephthalate (PET) [2]. Therefore, achieving the industrial synthesis of isopentanol is of paramount importance.

Currently, the industrial production of isopentanol relies primarily on, chemical synthesis, which is hampered by several challenges, including complex production equipment, stringent reaction conditions, high manufacturing costs, and significant environmental impact [5]. Microbial fermentation offers a novel approach to producing branched-chain higher alcohols. This method requires no fossil fuels, operates under milder conditions, achieves high conversion rates, and produces low levels of pollution. Moreover, it is also in line with the strategic goal of sustainable development and holds considerable potential for future growth.

There are three known biosynthesis pathways for isopentanol in microbial cell factories: the catabolic process (Ehrlich pathway), the pyruvate synthesis pathway (Harris pathway), and the mevalonate (MVA) pathway [[6], [7], [8], [9]]. By introducing the last two stages of the Ehrlich pathway, enhanced synthesis of isopentanol has now been accomplished in several chassis [[10], [11], [12], [13], [14], [15]].

*Saccharomyces cerevisiae* naturally possesses the Ehrlich pathway and exhibits a higher tolerance to alcohol concentrations, making it a more suitable cell factory for isopentanol production. In *S. cerevisiae*, the upstream pathway from pyruvate to α-isopropylmalate (α-IPM) is in both mitochondria and cytoplasm, while the downstream pathway from α-IPM to isopentanol is localized in the cytoplasm. This spatial separation hinders the efficient participation of the intermediate α-IPM in downstream processes. To overcome the limitations imposed by subcellular compartmentalization, Yuan et al. [12] overexpressed the mitochondrial α-IPM transporter protein-encoding gene *OAC1*, resulting in a 19.9–34.4% increase in isopentanol production. Similarly, overexpression of the yeast mitochondrial pyruvate carriers (MPC) has been successfully shown to enhance the mitochondrion-based biosynthesis of acetoin in *Candida glabrata* [16]. However, mitochondrial transport proteins involving α-KIV and valine remain unidentified [17,18], significantly limiting the use of compartmentalization strategies in constructing efficient cell factories [19]. Previous studies have primarily focused on modifying key genes in known pathways which, while effective, often overlook the potential roles of putative functional genes. Differing from prior approaches, Wang et al. [20] developed a novel molecular device for base editing at random genomic loci (named Helicase-AID), enabling C to T editing and the accumulation of random single-base mutations across the genome. This method, which increased the mutation rate by 1.68 × 10^3^-fold compared to wild-type strains, could be used to construct mutation pools encompassing nearly all functional genes. Beyond metabolic pathway engineering, effective screening methods are crucial for screening high-yielding strains and optimizing production pathways. Genetically encoded biosensors, which convert biological responses into observable signals (such as color change, luminescence, or fluorescence), have shown powerful advances in high-throughput screening. Zhang et al. [21] developed a novel biosensor for monitoring the metabolic fluxes of the branched-chain higher alcohol pathway by utilizing the yeast endogenous regulator Leu3p.

In this study, we coupled a genome-wide continuous perturbation tool with a biosensor-based high-throughput screening strategy, leading to the construction of five high-yielding isopentanol mutant strains. We then conducted a systematic transcriptomic analysis of the isopentanol synthesis pathway, branching competition pathways, glycolysis pathway, and ethanol fermentation pathway. Further analysis of co-differentially expressed genes (co-DEGs) in high-yield mutant strains allowed us to identify key targets outside the primary synthetic pathway that could enhance isopentanol synthesis. Based on these findings, we offer new insights into the metabolic network of branched-chain higher alcohols and lay the groundwork for developing clean and efficient isopentanol-producing cell factories.

## 2 Material and method

### 2.1 Plasmid and strain construction

DNA construction was performed using standard restriction-enzyme digestion and ligation cloning*. E. coli* DH5α was used for routine transformations and plasmid production. Endogenous *S. cerevisiae* genes were amplified from the genomic DNA of CEN.PK2–1C by PCR using a forward primer containing a *Nhe*I restriction recognition site and a reverse primer containing an *Xho*I restriction recognition site (Supplementary Table S3). This enabled the subcloning of PCR-amplified genes into pYZ92 plasmids. All of the constructed plasmids (Supplementary Table S2) were verified by DNA sequencing. *S. cerevisiae* strains used were derived from CEN.PK2–1C (*MATα ura3-52 trp1-289 leu2-3,112 his3-1 MAL2-8c SUC2*). The strains obtained through modification and evolution are listed in Supplementary Table S1. Deletions of *HOM3*, *DIP5*, *ROX1*, and *ATG41* were obtained using PCR-based homologous recombination. The DNA fragment containing the Lox71-*URA3*-Lox66 cassettes and the upstream and downstream 1500 bp homology arms of the target knockout genes. The gel-purified PCR fragments were transformed using the lithium acetate method [22]. Cells transformed using *URA3* labeling were inoculated and cultured on SC-URA plates supplemented with 2% glucose.

### 2.2 Culture condition

*E. coli* strains were cultivated at 37 °C in Luria-Bertani medium (5 g/L yeast extract, 10 g/L tryptone, and 10 g/L NaCl) with or without 50 μg/mL of the indicated antibiotics as required. For LB agar plates, 20 g/L agar was added to the medium. *S. cerevisiae* was cultured at 30°C on either YPD medium (10 g/L yeast extract, 10 g/L peptone, 0.15 g/L tryptone, and 20 g/L glucose) or synthetic complete (SC) medium (1.5 g/L yeast nitrogen source medium without amino acids and ammonium sulfate, 5 g/L ammonium sulfate, 36 mg/L myoinositol, and 2 g/L amino acid mixture). Supplementation of the SC medium with 2%, 10%, or 15% glucose, or a non-fermentable carbon source 2% raffinose, is required under specific conditions. For SC-URA medium (amino acid mixture without URA), 2% glucose or raffinose was added as the carbon source. Agar plates were prepared with the addition of 2% agar powder.

### 2.3 Construction of genome-wide perturbation library

The blank plasmids pRS416 and pMCM5-AID (Helicase-AID tool plasmid, provided by Dr. Changhao Bi) were transformed into the strain QXy1 respectively. The colonies were first inoculated into SC-URA medium supplemented with 2% glucose and cultured at 30°C overnight. The cultures were then diluted 10-fold and transferred to 2% raffinose medium, followed by overnight incubation. The cells were subsequently diluted 20-fold into 0.02% galactose and 2% raffinose medium to induce pMCM5-AID expression. After the editing process, pMCM5-AID was further removed to prevent the destabilizing effects of continuous perturbation on subsequent detection.

5-fluoroorotic acid (5-FOA) was used to remove pMCM5-AID plasmid from an *S. cerevisiae* strain. Cells were grown overnight in a YPD liquid medium, then spread onto YPD agar plates containing 1 mg/mL 5-FOA. Following this, the strains were streaked on YPD and SC-URA plates with 2% glucose to confirm the elimination of the editing plasmid.

### 2.4 Flow Cytometry/FACS

Fluorescence measurements and FACS were performed on samples in the mid-exponential growth phase. Single colonies from agar plates or yeast transformation libraries were diluted 1:100 and cultured overnight until stationary phase in synthetic complete (SC) medium supplemented with 2% glucose. The overnight cultures were then diluted 1:100 into the same fresh medium and incubated until mid-exponential growth (12–13 h after inoculation). The culture was diluted using SC with 2% glucose to achieve an OD_600_ of approximately 0.8 for subsequent fluorescence-activated cell sorting (FACS). All cells for flow cytometry analysis were diluted to an OD_600_ approximately 0.2 using sterile PBS.

Flow cytometry analysis and sorting were performed using BD FACSAria Fusion SORP with FACSDiva software. yEGFP detection was carried out at an excitation wavelength of 488 nm and emission wavelength of 510 nm. Cells were gated based on forward scatter (FSC) and side scatter (SSC) signals to exclude cellular debris and aggregates. Each sample was measured with 50,000 events, resulting in the top 1% of cells exhibiting high levels of GFP fluorescence. The sorted cells were collected into 1 mL of SC with 2% glucose medium, and 50 μL was transferred to the matching agar plates and incubated at 30°C to obtain random colonies for isopentanol production analysis. The remaining sorted cells (∼950 μL) were transferred to 5 mL of SC with 2% glucose in the liquid medium, cultivated until stable growth, and then dilution (1:100) for the next round of sorting. All data were obtained using FlowJo X software analysis.

### 2.5 Isopentanol fermentation production

High cell density fermentations were taken out on sterile 24-well microtiter plates at 30°C and 200 rpm. Random colonies obtained by FACS were inoculated in 1 mL of SC or SC-URA with 2% glucose medium for overnight growth. Then, the cells were diluted 100-fold and transferred in the same medium in a new 24-well plate. After 20 h, the plates were centrifuged at 234 *g* for 5 min with the supernatant discarded, and the cells were resuspended in 1 mL SC or SC-URA medium supplemented with 15% or 10% glucose. The plates were covered with sterile adhesive SealPlate films (Catalog No. STR-SEAL-PLT, CA) and fermented for 48 h under the same conditions with shaking. SealPlate membranes ensure micro-oxygenated conditions in each well while preventing medium evaporation and cross-contamination between wells. We continuously monitored at 1 h, 12 h, 24 h, 36 h, and 48 h intervals during microaerobic fermentation. Plates were then centrifuged for 5 min at 234 *g*, and the supernatant from each well was analyzed using High-Performance Liquid Chromatography (HPLC). The glucose, ethanol, glycerol, isobutanol, and isopentanol concentrations were detected using an Aminex HPX-87 ion exchange column at a flow rate of 0.6 mL/min at 55°C with 5 mM sulfuric acid as the mobile phase and a refractive index detector (RID). A standard curve was constructed based on the peak areas of isopentanol standards at various concentrations and quantified by comparing it with the peak areas detected in the fermentation broth.

### 2.6 Transcriptomics analysis

For transcriptome analysis, five mutants were grown in SC medium supplemented with 2% glucose at 30°C and 200 rpm. Cells at the late mid-exponential stage (16 h) were collected for RNA isolation. The RNA preparation, library creation, and sequencing were carried out by MAGIGENE (Tianjin, China). Wayne plots, volcano maps, and enriched KEGG pathways plots were drawn online using the Lianchuan BioCloud platform, and common differential gene analysis was performed for genes with |log2FC| ≥ 1 and P < 0.05.

### 2.7 Statistical analysis

A two-sided Student's t-test was performed using GraphPad Prism to determine the statistical significance of differences in titers between strains. Probability values (P-values) less than or equal to 0.05 were considered sufficient to reject the null hypothesis (that the means between the two samples are the same) and accept the alternative hypothesis (that the means between the two samples are different).

## 3 Results

### 3.1 Screening of high-yielding isopentanol mutants from genome-wide perturbation libraries using biosensor

**Fig. 1:**
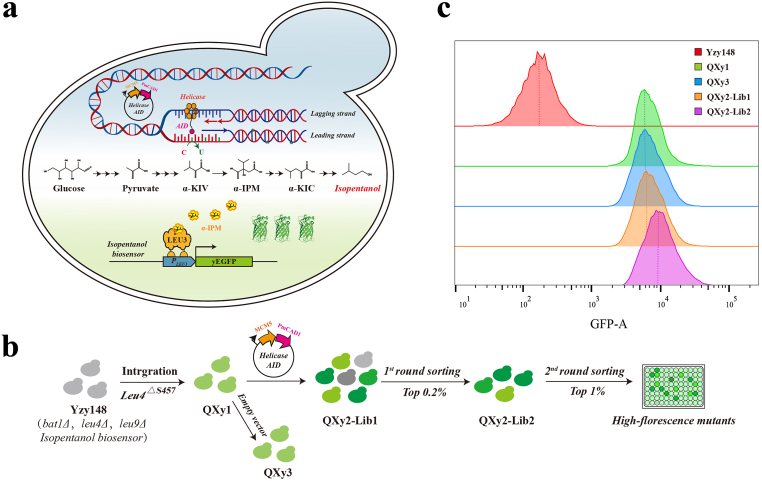
Coupling genome-wide perturbation tool and genetically encoded biosensors in *S. cerevisiae.* (a) The inducible base editing tool (Helicase-AID) consists of helicase MCM5 and cytosine deaminase PmCAD1. This tool continuously introduces C to T mutations (indicated by the green star) in the leading strand. The isopentanol biosensor is regulated by the Leu3p-regulated LEU1 promoter (*P*_*LEU1*_) and drives the expression of yeast-enhanced green fluorescent protein (yEGFP), which is activated by α-IPM. α-KIV, α-ketoisovalerate; α-IPM, α-isopropylmalate; α-KIC, α-ketoisocaproate. (b) Workflow of high-throughput screening using the isopentanol biosensors. (c) Flow cytometry analysis of the global perturbation library QXy2-Lib1 (orange) and QXy2-Lib2 (purple). Control strains include a synthetic pathway-incomplete strain (Yzy148, red), the isopentanol-producing strain QXy1 (green), and a strain with an empty vector (QXy3, blue). Cell populations were normalized to the mode for comparison.

Bridging perturbation library construction and high-throughput screening is an efficient strategy for obtaining high-yielding strains in metabolic engineering. We introduced genome-wide perturbations to an isopentanol-producing strain QXy1 (*bat1Δ*, *Leu4*^*ΔS457*^, *leu9Δ*, and isopentanol biosensor), which harbors a chromosome-integrated isopentanol biosensor [21] (Fig. 1a). The perturbation libraries were constructed using an inducible base editing tool, Helicase-AID, which continuously performs C to T editing and accumulates single base mutations at random genomic loci [20]. The isopentanol biosensor responds to α-IPM, the precursor of isopentanol, and reflects the synthetic capability of this branched-chain higher alcohol [21].

**Fig. 2:**
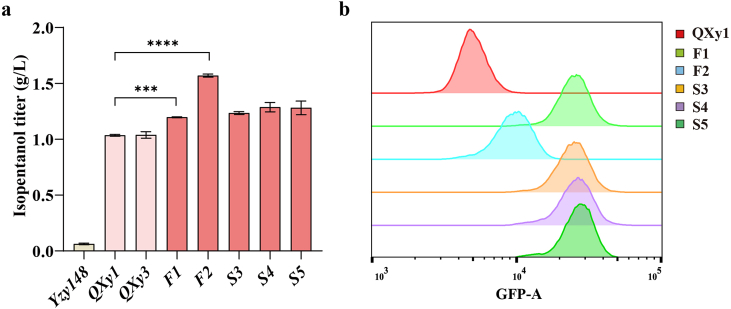
Isopentanol production and flow cytometry analysis of mutant strains. (a) Isopentanol titers after 48 h of fermentations in 15% glucose of isolated mutant strains and the control strains (∗∗∗P ≤ 0.001, ∗∗∗∗P ≤ 0.0001). Control strains include Yzy148, QXy1, and QXy3. (b) Flow cytometry analysis of five high-yielding mutants containing F1 (green); F2 (blue); S3 (orange); S4 (purple); and S5 (dark green), compared to control strains QXy1 (red).

The strain QXy1, prior to perturbation evolution, produced 1.04 ± 0.009 g/L of isopentanol, with the biosensor exhibiting a stronger fluorescence signal compared to the control strain (YZy148), which has an incomplete isopentanol synthesis pathway. Meanwhile, the fluorescence signal of QXy3, carrying the empty vector, was similar to that of QXy1 (Fig. 1b and c). A small population in the genome-wide perturbation library (QXy2-Lib1) showed a slightly increased fluorescence signal. We sorted the top 0.2% of QXy2-Lib1 using fluorescence-activated cell sorting (FACS) to obtain a refined library, QXy2-Lib2. The GFP median fluorescence intensity (MFI) of QXy2-Lib2 was significantly higher than that of both QXy2-Lib1 and QXy1. In the subsequent round of sorting, we collected single cells from the top 1% of QXy2-Lib2 into 96-well plates for fermentation and growth validation (Fig. 1b and c). This process resulted in the identification of two strains, F1 and F2, which achieved isopentanol titers of 1.20 ± 0.004 g/L and 1.57 ± 0.014 g/L, respectively. These titers represent increases of 15.6% and 51.7% compared to the control strain QXy1, and their MFI showed a significant enhancement (Fig. 2a, Supplementary Fig. 1a).

**Fig. 3:**
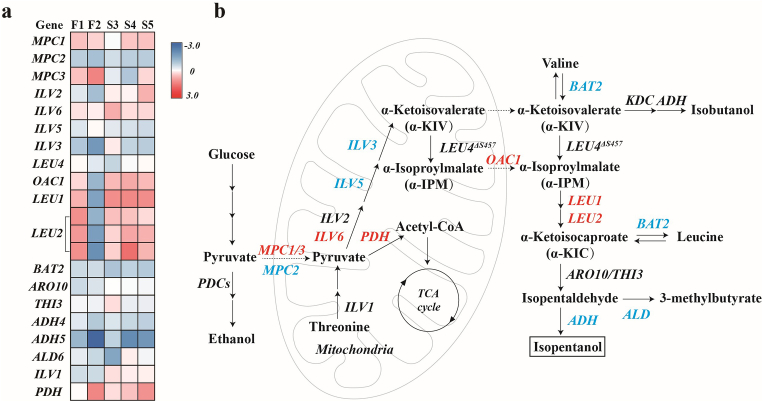
Transcriptome analysis of isopentanol synthesis pathways. (a) Log2 fold change (Log2FC) values of genes involved in the synthesis pathway starting from pyruvate, compared to the control strain QXy1 across the five high-yielding mutant strains. (b) Isopentanol synthesis pathway in *S*. *cerevisiae*. This pathway spans two compartments: the mitochondrial and cytoplasmic matrix. Red indicates the upregulated transcription DEGs, and blue indicates the downregulated transcription DEGs.

To further enhance isopentanol production, we performed a second round of perturbation screening cycles using F1 and F2 as starting strains (Supplementary Fig. 1b). This yielded three additional higher-yielding mutant strains: S3, S4, and S5, which achieved isopentanol titers of 1.24 ± 0.013 g/L, 1.29 ± 0.042 g/L, and 1.28 ± 0.061 g/L, respectively, representing increases of 19.3%, 24.2%, and 23.7% over QXy1 (Fig. 3a). Notably, these three strains (S3, S4, and S5), obtained from the second round of editing and evolution, were all derived from the F1 strain, showing improvements of 3.4%, 7.5%, and 7.0% over F1, respectively. All five mutants showed a significant overall increase in fluorescence signal compared to QXy1 (Fig. 2b), demonstrating the effectiveness and sensitivity of the isopentanol biosensor.

**Fig. 4:**
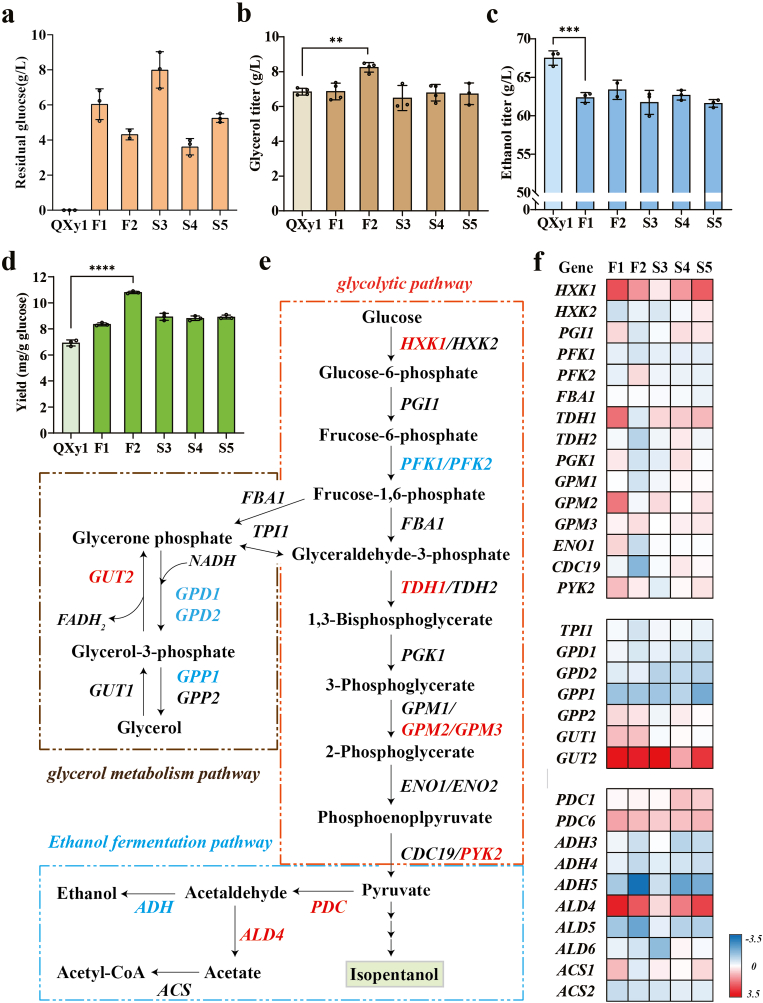
Transcriptome analysis of genes involved in glycolytic, glycerol metabolism, and ethanol fermentation pathways. (a) Residual glucose concentration, (b) Glycerol titer, (c) Ethanol titer, and (d) Isopentanol yield of the five mutants after 48 h of fermentation. The control strain QXy1 is the isopentanol-producing strain prior to the genome-wide perturbation (∗∗P ≤ 0.01, ∗∗∗P ≤ 0.001,∗∗∗∗P ≤ 0.0001). (e) Schematic of the glycolysis pathway, glycerol metabolism pathway, and ethanol fermentation pathway in *S. cerevisiae*. Red indicates upregulated transcription DEGs; Blue indicates downregulated transcription DEGs. (f) Log2FC values of genes involved in the glycolysis pathway, glycerol metabolism pathway, and ethanol fermentation pathway compared to the control strain QXy1 in the five high-yielding mutant strains. Red indicates upregulated transcription DEGs; Blue indicates downregulated transcription DEGs.

### 3.2 Systematic transcriptome analysis of high-yielding strains

To identify the key targets influencing isopentanol production, we conducted a systematic transcriptome analysis on the five high-yielding mutant strains (Supplementary Fig. 4). Firstly, we selected mutant strains F2 (the highest-yielding strain) and S3 (a representative strain of the S1 lineage) for genomic mutation analysis. Strains F2 and S3 had 1325 and 1276 SNPs, respectively, with C-to-T mutations, consistent with the mutation patterns predicted by the base mutation tool, accounting for 26.6% and 27.0% of the total SNPs (Supplementary Table S5). These findings suggest that effective mutations at the genomic level contribute to the high yield in these strains. Subsequently, we focused on key metabolic pathways through systematic transcriptome analysis to further unravel the metabolic network underlying isopentanol production.

Based on this, we first focused on the genes involved in the isopentanol synthesis pathway starting from pyruvate entering mitochondria. Mutant strains F1, S3, S4, and S5 showed similar transcriptional trends (Fig. 3a). In contrast, the majority of the genes in the F2 strain showed opposing transcriptional patterns, aligning with the screening results that indicated S3, S4, and S5 are derived from strain F1. Notably, genes encoding the mitochondrial pyruvate transporter proteins *MPC1* and *MPC3* were slightly transcriptionally upregulated in mutant strains F1 and F2, while *MPC2* was transcriptionally downregulated in all mutant strains (Fig. 3a and b). This finding is consistent with previous research indicating that the Mpc1p and Mpc2p complex function as active mitochondrial pyruvate carriers under aerobic conditions, while the Mpc1p and Mpc3p complex serve as low-activity transporters under anaerobic conditions [23]. Additionally, *OAC1*, which encodes the mitochondrial transporter protein for the critical intermediate α-IPM, also showed transcriptional upregulation (Fig. 3a and b), potentially directing more carbon flow toward isopentanol production [12].

In the upstream pathway of isopentanol synthesis of mutant strain F1 and its series strains, *ILV6*, which encodes a regulatory subunit of acetolactate synthase, demonstrated transcriptional upregulation despite being subject to feedback inhibition by branched-chain amino acids [24]. In contrast, *ILV5* and *ILV3* showed transcriptional downregulation (Fig. 3a), possibly to balance protein resources and intermediates accumulation within the mitochondria, thereby creating a homeostatic state that benefits yeast survival. In the downstream synthesis pathway, both *LEU1* and *LEU2* were upregulated in the mutant strains, except for F2 (Fig. 3a). This upregulation facilitated the efficient channeling of the intermediate α-IPM into the downstream pathway for isopentanol production. In contrast, the *ADH* gene showed minor transcriptional downregulation (Fig. 3b), potentially due to other upregulated genes competing for protein resources in the cytoplasm.

We also observed that the key genes involved in the branched-chain amino acid branching pathway and the methyl butyrate competition pathway, *BAT2*, and *ALD*, were transcriptionally downregulated, which could decrease the loss of carbon flow in the isopentanol synthesis pathway (Fig. 3a and b). Conversely, the *PDH* gene exhibited transcriptional upregulation, potentially competing with the target product for the precursor pyruvate, but generating more acetyl-CoA involved in the TCA cycle.

We continuously monitored the fermentation process of the mutant strain. None of the mutant strains showed significant growth defects during fermentation (Supplementary Fig. 2a). Fermentation data indicated that the high-yielding mutants exhibited lower glucose consumption and ethanol production compared to QXy1, suggesting higher sugar utilization efficiency and reduced competition for ethanol production (Fig. 4a and c). In particular, mutant strain F2 showed a considerable increase in glycerol production, while the other mutant strains did not differ significantly from the control strain (Fig. 4b, Supplementary Fig. 2c). The isopentanol yield of all mutants was significantly higher than that of the control strain QXy1, with the F2 mutant reaching 10.78 ± 0.074 mg/g glucose, representing a 56.2% increase over QXy1 (Fig. 4d). Interestingly, the fermentation medium supplemented with 10% glucose resulted in a notable increase in the total yield, reaching 14.04 ± 0.251 mg/g for strain F2, which exceeded the highest yield reported so far (Supplementary Table S4, Supplementary Fig. 3d). Moreover, all mutant strains exhibited lower titers of isobutanol, which facilitates the reduction of the cost of purifying isopentanol by distillation in the industry (Supplementary Fig. 2e).

We further analyzed transcriptional differences in key genes of glycolysis, glycerol metabolism, and ethanol fermentation pathways. Transcriptome data revealed that strain F2 continued to exhibit trends opposite to the other four mutant strains, with most glycolysis-related genes showing transcriptional downregulation, correlating with the lowest glucose consumption observed in strain F2 after fermentation (Fig. 4e and f). All high-yielding strains showed transcriptional upregulation of *HXK1*, the first key gene in the glycolytic pathway. Further transcriptome analysis of genes involved in the glycerol synthesis pathway revealed an overall trend of transcriptional downregulation in the mutant strains (Fig. 4f). Interestingly, despite the downregulation of glycerol biosynthesis-related genes, the F2 strain exhibited higher glycerol levels than the control strain QXy1 (Fig. 4b). The glycerol metabolic pathway is regulated by redox balance under anaerobic conditions, and the key enzyme Gut2p (glycerol-3-phosphate dehydrogenase) is involved in the reaction process to generate FADH_2_ into the mitochondrial respiratory chain to participate in energy metabolism. All mutant strains showed significant transcriptional upregulation of the *GUT2* gene, which maintained cellular redox and energy homeostasis and supplied more cofactors involved in isopentanol biosynthesis. Furthermore, we examined the genes associated with the observed reduction in ethanol production. The essential gene *ADH*, responsible for ethanol conversion, showed transcriptional downregulation, while *ALD4*, a key gene in the competitive pathway, exhibited considerable transcriptional upregulation, contributing to the lower ethanol titer (Fig. 4e and f). We also observed a slight transcriptional upregulation in the pyruvate dehydrogenase-encoding genes *PDC1* and *PDC6*, suggesting that the competition for the common precursor pyruvate was not reduced in the mutants, although overall ethanol synthesis was downregulated.

We further explored the uniqueness of the transcriptional differences in the F2 strain and found that mutant strains F2 and S3 grew at a slower rate compared to the control strain QXy1 after 16 h of incubation (Supplementary Fig. 5a). Additionally, the residual sugar concentration in the mutant strain F2 was significantly higher than that of QXy1, in contrast to the F1 series mutant strains, which showed almost no residual sugar (Supplementary Fig. 5b). This observation may explain the notable transcriptional differences between the F2 strain and the F1 series of mutant strains.

### 3.3 Functional analysis of co-expressed differentially expressed genes (co-DEGs)

**Fig. 5:**
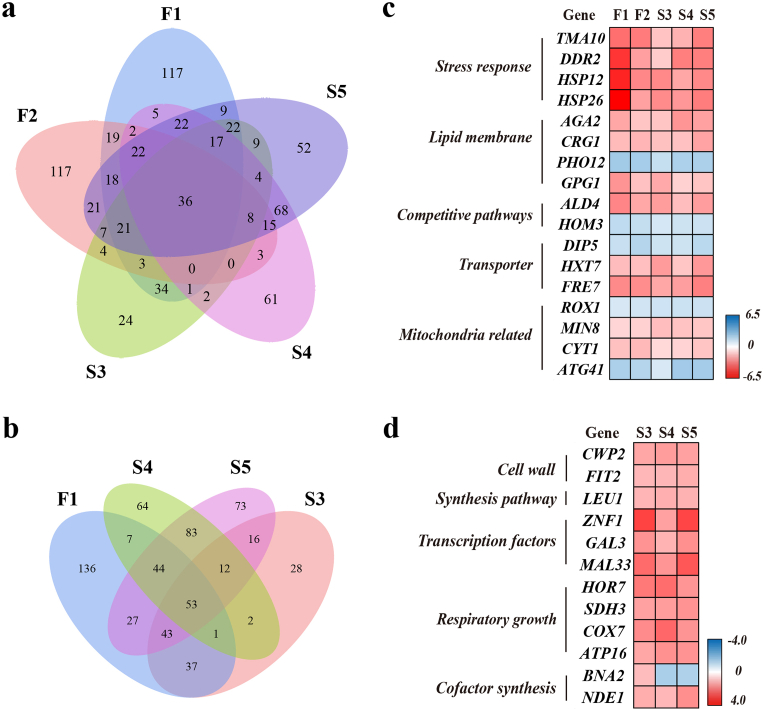
Transcriptome analysis of co-DEGs in high-yielding mutants. (a–b) Venn diagram of DEGs: (a) the five mutant strains and (b) F1 and its derived mutant strains (S3, S4, S5), with genes meeting the criteria of |log2FC| ≥ 1 and P < 0.05. (c–d) Transcriptome analysis of co-DEGs: (c) the five mutant strains and (d) F1 and its derived mutant strains (S3, S4, S5). Red indicates the upregulated transcription DEGs, while blue indicates the downregulated transcription DEGs.

We screened five mutant strains for co-expressed DEGs (co-DEGs) to identify potential key targets and expand the isopentanol anabolic network. Using |log2FC| ≥ 1 and P < 0.05 as reference values, we initially identified 36 co-DEGs. After excluding genes with low expression levels, 17 co-DEGs were retained for detailed analysis (Fig. 5a). These co-DEGs were categorized into five groups based on their biological functions (Fig. 5c).

(1) Stress response protein-related genes, *TMA10*, *DDR2*, *HSP12*, and *HSP26* all showed transcriptional upregulation, indicating enhanced cellular resistance to stress caused by perturbation. *TMA10* and *DDR2* are involved in DNA replication [25] and environmental stress responses, respectively. Hsp12p and Hsp26p are both members of the heat shock protein family. Hsp12p maintains the stability of membrane organization under oxidative, hyperosmotic, and thermal stress situations [26], while Hsp26p suppresses the aggregation of unfolded proteins [27]. (2) Plasma membrane proteins, Aga2p, Crg1p, and Pho12p, are crucial for maintaining the stability of the cell and the mitochondrial membranes, ensuring the normal cellular phenotype. Gpg1p, a G protein subunit, is involved in transmembrane signaling and regulates the growth and development of pseudo hyphae [28]. The pronounced transcriptional differences in the genes they encode are essential for maintaining the normal structure and function of cell membranes in the mutant strains. (3) Competitive pathway-related genes, *ALD4* and *HOM3*, are involved in the fermentative ethanol pathway and the common pathway for methionine and threonine biosynthesis, respectively [29]. Their transcriptional downregulation can reduce the utilization of carbon flux in these competing pathways. (4) Transporter proteins Dip5p mediates the transmembrane transport of acidic amino acids [30]. The transcriptional downregulation of this gene has a similar role to *HOM3*. In contrast, *HXT7*, encoding a high-affinity glucose transporter, was transcriptionally upregulated, enhancing the efficient delivery of fermentation carbon sources. *FRE7*, which encodes an iron reductase that decreases iron bound in iron carriers before it is taken up by transporter proteins [31], is not directly linked to isopentanol synthesis but plays a role in iron metabolism. (5) Mitochondria-related genes, *ROX1*, *MIN8*, and *CYT1* are involved in regulating the aerobic respiratory chain within the inner mitochondrial membrane. Except for *ROX1*, which acts as a transcriptional repressor under low oxygen conditions [32], these genes were transcriptionally upregulated, consistent with their functions. The transcriptional downregulation of *ROX1* aligns with the microaerobic fermentation conditions. Min8p balances the ratio of complex IV to ATP synthase to prevent cell death [33]. while Cyt1p, a major component of complex III, plays a direct role in electron transfer [34]. Additionally, *ATG41* related to mitochondrial autophagosome formation and respiratory growth [35], was strongly downregulated in all mutants.

This analysis implies that efficient glucose uptake and the suppression of competitive pathways are potential key factors in enhancing isopentanol production. Additionally, mitochondrial function, particularly the activity of the aerobic respiratory chain and the rate of autophagy, plays a significant role in optimizing isopentanol yield.

Strains S3, S4, and S5 were all derived from the second round of evolution of strain F1, suggesting that the co-DEGs in these three mutant strains likely contain the key targets that we are seeking. Based on this hypothesis, we screened these four mutant strains for co-DEGs, using |log2FC| ≥ 1 and P < 0.05 as the threshold. Strain S3, S4, and S5 share 12 co-DEGs based on the F1 strain, which we classified into five groups according to their biological functions (Fig. 5b and d).

(1) Cell wall-related genes, *CWP2* and *FIT2*, exhibited a tendency toward transcriptional upregulation, which favors the maintenance of cell wall stability. Cwp2p can be covalently linked to mannoprotein to stabilize the cell wall [36]. Although Fit2p is not a direct component of the cell wall, it is involved in iron retention within the cell wall [37]. (2) Synthesis pathway-related gene *LEU1*, an essential gene in the isopentanol synthesis pathway, showed transcriptional upregulation. (3) Transcriptional activators, *ZNF1*, *GAL3*, and *MAL33*, were identified as being considerably transcriptionally upregulated. Notably, *ZNF1* showed the highest level of upregulation and is involved in promoter binding for respiration, gluconeogenesis, and glyoxylate shunting as a zinc cluster transcription factor [38]. *GAL3* and *MAL33* encode transcriptional regulators of galactose and maltose, respectively. The global evolutionary tool, Helicase-AID, utilized in this study requires galactose-induced initiation, likely explaining the observed upregulation of *GAL3*. (4) Aerobic growth-related genes in all four co-DEGs screened showed a trend of transcriptional upregulation, with *HOR7* increasing the stress resistance of cells in high Ca^+^ and high salt environments. *SDH3*, *COX7*, and *ATP16* are involved in aerobic respiration: Sdh3p and Cox7p function as members of Complex II and Complex IV in the electron transport chain, respectively. While Atp16p, a subunit of the mitochondrial F_1_–F_0_ ATP synthase, is directly involved in ATP synthesis [39]. (5) Cofactor-related genes, *BNA2* and *NDE1*, exhibited different transcriptional trends. *BNA2* was the only co-DEGs that showed varying trends in strains S3, S4, and S5. Bna2p is required for the initiation of NAD^+^ biosynthesis from tryptophan [40], and NAD^+^ acts as a cofactor in the Leu2p-catalyzed reaction for the synthesis of the isopentanol precursor α-KDC. However, *BNA2* was transcriptionally upregulated in strain S3 and downregulated in strains S4 and S5. Conversely, *NDE1* was consistently upregulated in all three mutant strains. Nde1p, an external mitochondrial NADH dehydrogenase, catalyzes the oxidation of cytoplasmic NADH [41], and its upregulation may provide more cytoplasmic NADH for the mitochondrial respiratory chain and isopentanol synthesis.

The analysis of co-DEGs in the second round of evolutionary mutants further confirms the potential involvement of the aerobic respiratory chain and ATP synthesis in establishing an effective isopentanol cell factory. Additionally, the auxiliary roles of transcriptional activators and cofactors, as well as the transcriptional differences observed in *HOR7*, *FRE7*, and *FIT2*, suggested that metal ions are also involved in isopentanol synthesis, potentially related to intermediary transporter proteins [31,37].

### 3.4 Functional validation of co-expressed differentially expressed genes (co-DEGs)

**Fig. 6:**
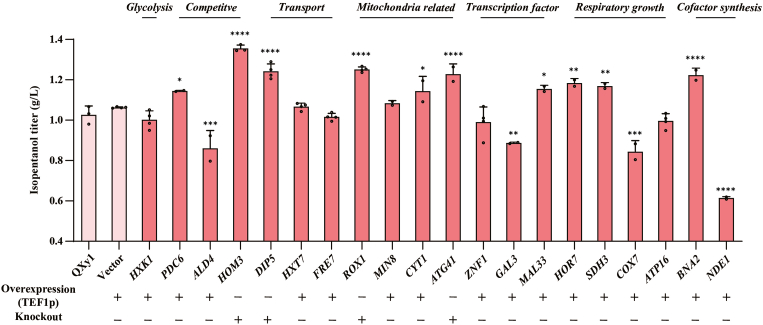
Overexpression or knockout validation of co-DEGs. Isopentanol titers were measured after 48 h of fermentation in 15% glucose of the verified strain. Control strains included QXy1 and a vector strain transformed with blank plasmid pYZ92 (using TEF1p as a promoter). "+" indicates overexpression and knockout validation of the corresponding genes in the QXy1 strain, respectively. ∗P ≤ 0.05, ∗∗P ≤ 0.01, ∗∗∗P ≤ 0.001,∗∗∗∗P ≤ 0.0001.

To further validate the functional validation of the identified co-DEGs associated with isopentanol production, we used TEF1p as a promoter to overexpress transcriptionally upregulated co-DEGs and employed homologous recombination to knockout transcriptionally downregulated co-DEGs (Fig. 6).

First, knocking out *HOM3* and *DIP5*, critical genes in the competitive amino acid synthesis pathway and transport protein, significantly increased isopentanol titers. Additionally, the mitochondrial autophagy gene *ATG41* and the inner mitochondrial membrane respiratory chain-related genes *ROX1*, *CYT1*, and *SDH3* were found to play potential roles in isopentanol synthesis.

Furthermore, overexpression of *HOR7*, which is involved in cellular resistance to environmental stress, enhanced isopentanol production. This suggests that *HOR7* contributes to maintaining robust cellular growth during production conditions. In addition, overexpressing *MAL33* and *BNA2* also moderately improved isopentanol synthesis, emphasizing the importance of transcription factors and cofactors in optimizing biosynthetic pathways.

In contrast, overexpression of *ALD4*, *GAL3*, *COX7*, and *NDE1* reduced isopentanol titers, likely due to metabolic imbalances or cellular stress from high promoter-driven expression levels. The strong correlation between the expression of these genes and efficient isopentanol production is undeniable. Modifications of other co-DEGs did not lead to significant changes in isopentanol production. This may be attributed to the disconnect between transcriptional changes and protein expression, or the fact that the observed transcriptional differences influence broader metabolic networks rather than single key genes.

Our findings demonstrate that inhibiting competitive amino acid synthesis pathways (*HOM3* and *DIP5*), enhancing mitochondrial aerobic respiratory chain function (*ROX1*, *CYT1*, *HOR6*, *SDH3*, and *COX7*), weakening autophagy (*ATG41*), and improving cofactor balance (*BNA2* and *NDE1*) are critical strategies for optimizing isopentanol production. These results highlight the importance of targeting these pathways to improve biosynthetic efficiency.

## 4 Discussion

Traditional metabolic engineering methods often employ the "push-pull-knock" strategy to modulate the synthetic pathway and increase the production of the target product [[42], [43], [44]]. However, global optimization beyond the primary synthesis pathway is also critical. For instance, Mukherjee et al. utilized a high-resolution CRISPR-interfering (CRISPRi) library to identify key genes that improve acetic acid tolerance in *S. cerevisiae* [45]. Similarly, Tang et al. identified genes that are highly sensitive to levulinic acid by constructing and screening a genome-wide knockout library combined with bioinformatics analysis, which is important for optimizing the conversion of lignocellulosic biomass to levulinic acid [46]. Without relying on rational engineering, we combined the genome-wide perturbation tool Helicase-AID with an isopentanol biosensor-based high-throughput screening approach. An effective combination of perturbed library construction and high-throughput screening methods can significantly reduce the screening time and enhance screening efficiency, making it a powerful strategy in metabolic engineering for obtaining high-yield strains. In this study, we employed Helicase-AID as a perturbation tool to greatly expand the size and coverage of mutation libraries. Unlike traditional approaches targeting known key enzymes or synthetic pathways, this method facilitates the construction of genome-wide perturbation libraries, enabling a more comprehensive exploration of potential key genes and metabolic networks.

This strategy increased isopentanol production to 1.57 ± 0.014 g/L in a fermentation medium supplemented with 15% glucose, and similarly improved the yield of isopentanol to 14.04 ± 0.251 mg/g glucose in a fermentation medium supplemented with 10% glucose, which exceeded the highest titer and yield reported to date [47]. This strategy effectively broadens the metabolic network of branched-chain higher alcohols in yeast at a genome-wide scale, and when paired with an effective screening procedure, it can yield numerous high-yielding evolved strains in under two rounds of screening. Nonetheless, this technique has its limitations. The F2 strain, which achieved the highest isopentanol titer, exhibited the smallest total fluorescence shift. We identified a single point mutation, Lys25Phe, in the *LEU4* gene within the genome of mutant strain F2. This mutation, resulting from a cytosine-to-thymine transition, aligns with the activity of the Helicase-AID base editing tool. The mutation in *LEU4*, which encodes the synthesis gene for the key component α-IPM, directly impacts the biosensor's functionality and likely accounts for the minimized fluorescence signal variation observed in the F2 mutant strain. Consequently, using GFP intensity as a screening marker based on the F2 strain was ineffective in identifying even higher-yielding mutations in subsequent rounds. While the genome-wide perturbation approach minimizes the risk of missing key targets, it also increases the likelihood of impairing the functionality of the isopentanol biosensor. Therefore, future strategies might consider integrating the biosensor at a later stage to mitigate the impact of the evolutionary tool. Notably, strains S3, S4, and S5 were derived from the F1 strain in the second round of editing and evolution. These results imply that genome-wide perturbations were highly effective, enabling the identification of high-yielding strains in just one round. However, a second round of mutations could potentially introduce more negative mutations other than positive mutations.

Transcriptomics, the systematic analysis of transcript levels within intracellular metabolic networks, is a powerful tool for identifying potential key targets for metabolic engineering. Wang et al. utilized comprehensive genome, transcriptome, and metabolome analyses of evolved *E. coli* adapted for BCHA tolerance and discovered that mutations in negative regulators RpoS improve tolerance in *E. coli* [48]. Similarly, the effect of α-keto acid decarboxylase knockout on higher alcohol synthesis in *S*. *cerevisiae* was examined through transcriptomic analysis, leading to the identification of 13 co-DEGs linked to higher alcohol production, along with the proposal of three genes with previously unknown functions (*HMRA2*, *SIR1*, and *SIR3*) [49].

In this study, we conducted transcriptome analysis of five high-yield mutant strains and observed that most genes in the F2 strains showed transcriptional trends opposite to those in F1 and its derivatives (including S3, S4, and S5). On the one hand, this may be due to differences in the expression of the central carbon pathway arising from the glucose utilization defects exhibited by the F2 strains. On the other hand, this phenomenon highlights the diversity within the genome-wide perturbation library while also allowing us to accurately target common differential genes with consistent trends.

We conducted transcriptome analysis of the mutant strain F2 with the highest isopentanol production. Compared to the control strain QXy1, 413 genes showed transcriptional upregulation and 240 genes showed transcriptional downregulation in strain F2 (Supplementary Fig. 6a). KEGG pathway enrichment analyses revealed substantial enrichment in pathways such as amino acid biosynthesis, the central carbon pathway of sugar metabolism, and glycerolipid metabolism in the F2 mutant strain. Notably, there was no significant enrichment in the branched-chain amino acids biosynthesis pathway, which is directly associated with isopentanol production. However, the enrichment of the central carbon pathway in the mutant strain ensures sufficient availability of the crucial precursor pyruvate, which facilitates the synthesis of the target product (Supplementary Fig. 6b).

Further analysis of all DEGs that met the criteria of |log2FC| ≥ 2.5 and P < 0.05 in the mutant strain F2 highlighted some biological functions (Supplementary Fig. 6c). Beyond the previously identified co-DEGs (including *HOM3*, *HSP12*, *ADH5*, and *FRE7*), no strong links were established between the functions of other DEGs and efficient isopentanol production. Instead, most DEGs were associated with normal cell growth and nucleic acid synthesis, consistent with the high proportion of ribosome-related genes identified in KEGG enrichment analysis. Consequently, we propose that transcriptome analysis of strains F2 alone is insufficient to identify efficient production targets. Instead, a shared analysis across mutant strains remains a more effective approach to pinpoint key determinants of isopentanol production.

We observed downregulation in the co-transcription of key competitive pathway genes *BAT2*, *ALD*, *ADH*, *HOM3*, and *DIP3*, which reduced the diversion of common precursors and contributed to the channeling of more carbon fluxes toward isopentanol synthesis. It has been shown that employing mutations with low-catalytic activity in branched-chain amino acid aminotransferases (*BAT1*, *BAT2*) can reduce competition for branched-chain amino acids, thereby promoting the synthesis of branched-chain higher alcohols. Our observation that *BAT2* showed downregulated transcription aligns with these previous findings.

The potential roles of transcription factors and oxidative phosphorylation in isopentanol production were highlighted in the co-DEG analysis. Transcription factors *ZNF1*, *GAL3*, and *MAL33* all showed transcriptional upregulation in the mutants. *ZNF1*, in particular, is considered a critical gene in the pentose phosphate pathway (PPP), where it maintains cofactor balance by providing NADPH for the production of branched-chain higher alcohols [38]. Recent studies have uncovered additional functions of Znf1p in isopentanol production, including enhancing yeast tolerance to branched-chain higher alcohols, regulating the electron transport chain, mediating the transcriptional upregulation of synthetic pathway, and maintaining energy and redox balance [50,51]. Although the overexpression of Znf1p in the strain we constructed did not influence isopentanol production, this outcome may be attributed to the strength of the promoter we used. In addition, the strong transcriptional upregulation of *GUT2*, a key gene in the glycerol degradation pathway, highlights its potential importance in maintaining redox homeostasis, which is critical for constructing an efficient isopentanol-producing cellular factory. Interestingly, the overexpression of *GAL3* and *MAL33* led to significant fluctuations in isopentanol production. This suggests that *MAL33* may have an unexplored link to the biosynthesis of branched-chain higher alcohols, warranting further investigation to clarify its role and underlying mechanisms.

Oxidative phosphorylation yields more energy compared to substrate-level phosphorylation. The electron transport chain (ETC) complex is crucial in aerobic respiration, driving the proton motive force to produce intracellular ATP by oxidizing NADH and FADH_2_ derived from glycolysis and the TCA cycle. Jung et al. applied a low-activity ETC mutant to increase the intracellular NADH/NAD^+^ ratio, thereby promoting the production of 2,3-BDO and isobutanol [52]. In contrast, our findings revealed that the associated genes *NDE1*, *SDH3*, *CYT1*, and *COX7* influence the efficient synthesis of isopentanol throughout the entire process, from cofactor supply to ATP production, in the high-yielding mutant strains. *NDE1* encodes an extra-mitochondrial NADH dehydrogenase, whose transcriptional upregulation can increase NADH supply. *SDH3*, *CYT1*, and *COX7* encode subunits of complexes II, III, and IV, respectively, which generate the proton motive force that ultimately drives ATP production. Meanwhile, we observed transcriptional differences in two regulators, *ROX1* and *MIN8*. Rox1p acts as a transcriptional repressor of the cytochrome C oxidase subunit *COX5*/*CYC7* under aerobic conditions [32], its transcriptional downregulation which could restore the expression efficiency of complex IV. Min8p, which can block the assembly of respiratory chain complex IV, undergoes hydrolysis when the complex IV assembly factor Rcf2p associates with the ATP synthase subunit Atp9p. This process coordinates the maturation of complex IV and ATP synthase, preventing an imbalance in their ratio that could otherwise lead to cell death [33]. Thus, while overexpression of *MIN8* did not directly increase isopentanol titer, we assume that the mutant strains did not show significant growth defects during fermentation is associated with a small upregulation of this gene. The overall high activity of oxidative phosphorylation provides additional energy that directly supports both strain growth and isopentanol production. Mitochondria play a critical role as the cell's powerhouse, supporting essential metabolic processes. Additionally, the biosynthetic pathway of branched-chain higher alcohols spans two compartments, the mitochondria and the cytoplasmic matrix. Thus, the knockout of *ATG41* effectively reduced the rate of mitochondrial autophagy and directly promoted isopentanol biosynthesis. In addition, the strong correlation exhibited by *BNA2* and *NDE1* and isopentanol also indicates that the NADH/NAD^+^ plays a direct role as a cofactor in the catalytic reaction for isopentanol synthesis and competes with ETC for precursor supply.

We emphasize the critical role of weakening competitive pathways, strengthening mitochondria including their aerobic respiratory chain, and maintaining intracellular cofactor balance in the efficient production of isopentanol. Furthermore, we identified 13 novel targets, broadening the intracellular metabolic network associated with branched-chain higher alcohol production. These findings open new avenues for the sustainable and cost-effective development of isopentanol-producing cell factories.

## 5 Conclusion

We present a strategy that integrates a genome-wide perturbation library with an isopentanol biosensor for high-throughput screening, resulting in the identification of five high-yielding mutant strains within a short timeframe. Notably, the F2 strain achieved an isopentanol titer of 1.57 ± 0.014 g/L and improved the glucose conversion rate for isopentanol production, yielding 14.04 ± 0.251 mg/g glucose (10% glucose), which surpasses the highest values reported to date in engineered *Saccharomyces cerevisiae* through mitochondrial compartmentalization. Through systematic transcriptome analysis and key target knockout or overexpression validation, we highlight the critical role of mitochondria, particularly oxidative phosphorylation and alongside transcriptional regulators, in constructing an efficient biofuel-producing cellular factory. These findings suggest that in addition to traditional metabolic strategies, such as enhancing anabolic pathways, attenuating branching pathways, and improving glucose uptake, mitochondrial function, and cofactor balance are key factors in optimizing isopentanol production. We also hypothesize potential roles for *GAL3*, *MAL33*, and *HOX7* in isopentanol synthesis, thereby expanding the intracellular metabolic network of branched-chain higher alcohols and providing new avenues for the development of environmentally friendly and cost-effective biofuels.

## CRediT authorship contribution statement

**Qi Xiao:** Writing – review & editing, Writing – original draft, Visualization, Validation, Software, Methodology, Investigation, Formal analysis, Data curation, Conceptualization. **Jingjing Shi:** Writing – original draft, Validation, Investigation, Formal analysis, Data curation. **Lixian Wang:** Methodology. **Guoping Zhao:** Supervision, Project administration, Funding acquisition, Conceptualization. **Yanfei Zhang:** Writing – review & editing, Supervision, Project administration, Investigation, Funding acquisition, Conceptualization.

## Declaration of competing interest

The authors declare that they have no known competing financial interests or personal relationships that could have appeared to influence the work reported in this paper.
