# Supplementary material for: Coupling genome-wide continuous perturbation with biosensor screening reveals the potential targets in yeast isopentanol synthesis network
Source: Synth Syst Biotechnol. 2024 Dec 30;10(2):452–62. doi: 10.1016/j.synbio.2024.12.010 (PMC11799893; doi:10.1016/j.synbio.2024.12.010)
Supplement: Multimedia component.1 [file mmc1.docx]

**Supplementary Information for:**

**Coupling genome-wide continuous perturbation with biosensor screening reveals the potential targets in yeast isopentanol synthesis network**

Qi Xiao^a, b, c #^, Jingjing Shi^a, b #^, Lixian Wang^a, b^, Guoping Zhao^b, d^, Yanfei Zhang^a, b *^

a Tianjin Institute of Industrial Biotechnology, Chinese Academy of Sciences, Tianjin 300308, China

b National Center of Technology Innovation for Synthetic Biology, Tianjin 300308, China

c College of Life Sciences, University of Chinese Academy of Sciences, Beijing, 101408, China.

d CAS-Key Laboratory of Synthetic Biology, CAS Center for Excellence in Molecular Plant Sciences, Institute of Plant Physiology and Ecology, Chinese Academy of Sciences, Shanghai, 200032, China.

# These authors contributed equally to this work.

*Corresponding author: Yanfei Zhang

Tianjin Institute of Industrial Biotechnology,

Chinese Academy of Sciences,

32 West 7th Avenue, Tianjin Airport Economic Area,

Tianjin, 300308, China

Phone office: +86 022-24828724

Fax: +86 022-84861926

Email: zhangyf@tib.cas.cn

**Supplementary Tables**

**Table S1.** Strain used in this study

| **Strain** | **Description** | **Genotype (Plasmid contents in parenthesis)** | **Source** |
| --- | --- | --- | --- |
| Yzy148 | *bat1Δ, leu4Δ, leu9Δ,*Isopentanol biosensor | CEN.PK2-1C (*MATα ura3-52 trp1-289 leu2-3,112 his3-1 MAL2-8c SUC2*), *bat1Δ, leu4Δ, leu9Δ,* Isopentanol biosensor (*his3::HIS3*-P_LEU1_-yEGFP-T_ADH1_) | [1] |
| QXy1 | Yzy148*,* cassette from pQX1 | Yzy148, *leu4*::P_GPD_-*LEU4^△S457^*-T_ADH1_ | This study |
| QXy2 | QXy1, pMCM5-AID | QXy1, *CEN URA3* plasmid (P_GAL1_-*MCM5-PmCDA1*- T_ADH1_) | This study |
| QXy3 | QXy1, pRS416 | QXy1, *CEN URA3* plasmid | This study |
| QXy4 | QXy1, *hom3Δ* | QXy1, *hom3*::Lox71-*URA3*-Lox66 | This study |
| QXy5 | QXy1, *dip5Δ* | QXy1, *dip5*::Lox71-*URA3*-Lox66 | This study |
| QXy6 | QXy1, *rox1Δ* | QXy1, *rox1*::Lox71-*URA3*-Lox66 | This study |
| QXy7 | QXy1, *atg41Δ* | QXy1, *atg41*::Lox71-*URA3*-Lox66 | This study |
| F1 | QXy2, Mutant strains | The first-round perturbation library | This study |
| F2 | QXy2, Mutant strains | The first-round perturbation library | This study |
| S3 | F1, Mutant strains | The second-round perturbation library from the F1 strain | This study |
| S4 | F1, Mutant strains | The second-round perturbation library from the F1 strain | This study |
| S5 | F1, Mutant strains | The second-round perturbation library from the F1 strain | This study |

**Table S2.** Plasmid used in this study

| **Plasmid** | **Description** | **Source** |
| --- | --- | --- |
| pRS416 | Amp^R^, CEN, URA3 | Lab stock |
| pMCM5-AID | Amp^R^, CEN, URA3, P_GAL1_-*MCM5-PmCDA1*- T_ADH1_ | [2] |
| pYZ92 | Amp^R^, CEN, URA3, P_TEF1_-MCS*-*T_ACT1_ | Lab stock |
| pYZ17 | AmpR, Lox71-kanMX -Lox66 gene-disruption cassette | Lab stock |
| pQX1 | Amp^R^, Leu4INT, P_GPD_-*LEU4^△S457^*-T_ADH1_ | This study |
| pQX2 | Amp^R^, Lox71-URA3*-*Lox66 gene-disruption cassette | This study |
| pQX118 | Amp^R^, CEN, URA3, P_TEF1_-*HXK1*-T_ACT1_ | This study |
| pQX119 | Amp^R^, CEN, URA3, P_TEF1_-*PDC6*-T_ACT1_ | This study |
| pQX120 | Amp^R^, CEN, URA3, P_TEF1_-*ALD4*-T_ACT1_ | This study |
| pQX121 | Amp^R^, CEN, URA3, P_TEF1_-*HXT7*-T_ACT1_ | This study |
| pQX122 | Amp^R^, CEN, URA3, P_TEF1_-*FRE7*-T_ACT1_ | This study |
| pQX123 | Amp^R^, CEN, URA3, P_TEF1_-*MIN8*-T_ACT1_ | This study |
| pQX124 | Amp^R^, CEN, URA3, P_TEF1_-*CYT1*-T_ACT1_ | This study |
| pQX125 | Amp^R^, CEN, URA3, P_TEF1_-*ZNF1*-T_ACT1_ | This study |
| pQX126 | Amp^R^, CEN, URA3, P_TEF1_-*GAL3*-T_ACT1_ | This study |
| pQX127 | Amp^R^, CEN, URA3, P_TEF1_-*MAL33*-T_ACT1_ | This study |
| pQX128 | Amp^R^, CEN, URA3, P_TEF1_-*HOR7*-T_ACT1_ | This study |
| pQX129 | Amp^R^, CEN, URA3, P_TEF1_-*SDH3*-T_ACT1_ | This study |
| pQX130 | Amp^R^, CEN, URA3, P_TEF1_-*COX7*-T_ACT1_ | This study |
| pQX131 | Amp^R^, CEN, URA3, P_TEF1_-*ATP16*-T_ACT1_ | This study |
| pQX132 | Amp^R^, CEN, URA3, P_TEF1_-*BNA2*-T_ACT1_ | This study |
| pQX133 | Amp^R^, CEN, URA3, P_TEF1_-*NDE1*-T_ACT1_ | This study |

**Table S3.** Oligonucleotides used in this study

| **Oligo Name** | **Sequence** | **Description** |
| --- | --- | --- |
| Xq-oli-440 | CTCGAGTAATTAAGCGCCAATGATACCAAG | NheI-ScHXK1 Primer-F |
| Xq-oli-441 | GCTAGCCATATGGTTCATTTAGGTCCAAAG | XhoI-ScHXK1 Primer-R |
| Xq-oli-442 | CTCGAGTAATTATTGTTTGGCATTTGTAGCG | NheI-ScPDC6 Primer-F |
| Xq-oli-443 | GCTAGCCATATGTCTGAAATTACTCTTGGAAAATAC | XhoI-ScPDC6 Primer-R |
| Xq-oli-444 | ATGGCTAGCATGTTCAGTAGATCTACGCTC | NheI-ScALD4 Primer-F |
| Xq-oli-445 | TTACTCGAGTTACTCGTCCAATTTGGCAC | XhoI-ScALD4 Primer-R |
| Xq-oli-446 | CTCGAGTAAATGTCACAAGACGCTGCTATTG | NheI-ScHXT7 Primer-F |
| Xq-oli-447 | GCTAGCCATTTATTTGGTGCTGAACATTCTC | XhoI-ScHXT7 Primer-R |
| Xq-oli-448 | ATGGCTAGCATGATTGAAGAAAGAGATTTGG | NheI-ScFRE7 Primer-F |
| Xq-oli-449 | TTACTCGAGCTAGTAGCCAAAACTCTCGC | XhoI-ScFRE7 Primer-R |
| Xq-oli-450 | CTCGAGTAATTAATCCTTATCTTTGGCAAGGAC | NheI-ScMIN8 Primer-F |
| Xq-oli-451 | GCTAGCCATATGAGACCAGCACAGTTACTG | XhoI-ScMIN8 Primer-R |
| Xq-oli-452 | ATGGCTAGCATGTTTTCAAATCTATCTAAACGTTG | NheI-ScCYT1 Primer-F |
| Xq-oli-453 | TTACTCGAGCTACTTTCTTGGTTTTGGTG | XhoI-ScCYT1 Primer-R |
| Xq-oli-454 | ATGGCTAGCATGGCCCGCAATAGACAAGC | NheI-ScZNF1 Primer-F |
| Xq-oli-455 | TTACTCGAGTTAAGGAAGCGCATCTACATCTTC | XhoI-ScZNF1 Primer-R |
| Xq-oli-456 | ATGGCTAGCATGAATACAAACGTTCCAATATTC | NheI-ScGAL3 Primer-F |
| Xq-oli-457 | TTACTCGAGTTATTGTTCGTACAAACAAGTACC | XhoI-ScGAL3 Primer-R |
| Xq-oli-458 | ATGGCTAGCATGACTTTAGTCAAGTATGCATG | NheI-ScMAL33 Primer-F |
| Xq-oli-459 | TTACTCGAGTTAAGGAATTATGTCGTCTTCATC | XhoI-ScMAL33 Primer-R |
| Xq-oli-460 | ATGGCTAGCATGAAGTTATCTCAAGTTGTTG | NheI-ScHOR7 Primer-F |
| Xq-oli-461 | TTACTCGAGTTAAATCAAAAAGGCCAAAGC | XhoI-ScHOR7 Primer-R |
| Xq-oli-462 | ATGGCTAGCATGTCTGCAATGATGGTCAAG | NheI-ScSDH3 Primer-F |
| Xq-oli-463 | TTACTCGAGTCATAAAGTTAATAAATAAGTACCGAG | XhoI-ScSDH3 Primer-R |
| Xq-oli-464 | ATGGCTAGCATGGCTAATAAAGTTATTCAACTAC | NheI-ScCOX7 Primer-F |
| Xq-oli-465 | TTACTCGAGCTATGCCTTCTTGGCTTTGA | XhoI-ScCOX7 Primer-R |
| Xq-oli-466 | ATGGCTAGCATGTTACGTTCAATTATTGGAAAG | NheI-ScATP16 Primer-F |
| Xq-oli-467 | TTACTCGAGCTATTTCAATACGGATTGTAGG | XhoI-ScATP16 Primer-R |
| Xq-oli-468 | ATGGCTAGCATGAGACCACTACCAGTGCT | NheI-ScBNA2 Primer-F |
| Xq-oli-469 | TTACTCGAGTCAATTTTTGTCTTCATTTTTAATGTCC | XhoI-ScBNA2 Primer-R |
| Xq-oli-470 | CTCGAGTAACTAGATAGATGAATCTCTACCC | NheI-ScNDE1 Primer-F |
| Xq-oli-471 | GCTAGCCATATGATTAGACAATCATTAATGAAAAC | XhoI-ScNDE1 Primer-R |
| Xq-oli-496 | TACGCTGCAGGTCGACAACCC | Lox71-URA3-Lox66-F |
| Xq-oli-497 | CTAGTGGATCTGATATCACCTATACCGTTCGTATAGC | Lox71-URA3-Lox66-R |
| Xq-oli-498 | TCCGTAAGATTATAGCTAATGAAGGC | ScHOM3_KO_Up_F |
| Xq-oli-499 | GGTTGTCGACCTGCAGCGTAGTAAAAGTTAAAAAAAATGAAAGCTTCTG | ScHOM3_KO_Up_R |
| Xq-oli-500 | GTATAGGTGATATCAGATCCACTAGATCCACCTTTCTTCTTCACTTTAATG | ScHOM3_KO_Down_F |
| Xq-oli-501 | ACAAGTTTGATAATATAGATGTGCCG | ScHOM3_KO_Down_R |
| Xq-oli-502 | GCCTCTGGTTTTCAAATTAATTAACC | ScDIP5_KO_Up_F |
| Xq-oli-503 | GGTTGTCGACCTGCAGCGTATACTTAGAGTTAGTTCTTTTTTTCCTG | ScDIP5_KO_Up_R |
| Xq-oli-504 | GTATAGGTGATATCAGATCCACTAGATGCCTTTTTAATAAAAGCCTTAGG | ScDIP5_KO_Down_F |
| Xq-oli-505 | AGATTATCTAAAGCCTAATTATGGAC | ScDIP5_KO_Down_R |
| Xq-oli-506 | TTAGTTGAGGAGGTAGCAGCG | ScROX1_KO_Up_F |
| Xq-oli-507 | GGTTGTCGACCTGCAGCGTATGTTGATTGTCTAACTGCGTTC | ScROX1_KO_Up_R |
| Xq-oli-508 | GTATAGGTGATATCAGATCCACTAGTTTTTTTTTTCCATTTCTTCTTTCCG | ScROX1_KO_Down_F |
| Xq-oli-509 | TTAGGGGAAACTGGCGTAAAG | ScROX1_KO_Down_R |
| Xq-oli-510 | CAAGGGTAAGAAAGTCACCC | ScATG41_KO_Up_F |
| Xq-oli-511 | GGTTGTCGACCTGCAGCGTATGTAAATAATTGTAATTGTATATTATTGTGTG | ScATG41_KO_Up_R |
| Xq-oli-512 | GTATAGGTGATATCAGATCCACTAGCGCATTTTACCACATCTCTAC | ScATG41_KO_Down_F |
| Xq-oli-513 | TGATGCAGATCGAGATACAG | ScATG41_KO_Down_R |
|  |  |  |

**Table S4.** Optimization of fermentation media

| **Strain** | **Total glucose**  **(g/L)** | **Isopentanol titer**  **(g/L)** | **Residual glucose**  **(g/L)** | **Glycerol titer**  **(g/L)** | **Ethanol titer**  **(g/L)** | **Yield**  **(mg/g glucose)** |
| --- | --- | --- | --- | --- | --- | --- |
| QXy1 | 150 | 1.04 ± 0.009 | n.d. | 6.91 ± 0.189 | 67.51 ± 0.935 | 6.90 ± 0.057 |
|  | 100 | 0.88 ± 0.039 | n.d. | 5.73 ± 0.113 | 48.54 ± 0.268 | 8.77 ± 0.394 |
| F1 | 150 | 1.20 ± 0.004 | 6.04 ± 0.879 | 7.02 ± 0.445 | 62.37 ± 0.643 | 8.31 ± 0.042 |
|  | 100 | 1.09 ± 0.132 | n.d. | 5.52 ± 1.183 | 46.42 ± 0.684 | 10.91 ± 0.083 |
| F2 | 150 | 1.57 ± 0.014 | 4.32 ± 0.312 | 8.38 ± 0.098 | 63.37 ± 1.255 | 10.78 ± 0.074 |
|  | 100 | 1.40 ± 0.025 | n.d. | 3.29 ± 0.020 | 32.58 ± 2.455 | 14.04 ± 0.251 |
| S3 | 150 | 1.24 ± 0.013 | 8.00 ± 1.038 | 6.48 ± 0.722 | 61.74 ± 1.565 | 8.69 ± 0.053 |
|  | 100 | 1.10 ± 0.012 | n.d. | 5.98 ± 0.083 | 48.14 ± 0.670 | 11.06 ± 0.122 |
| S4 | 150 | 1.29 ± 0.042 | 3.62 ± 0.465 | 6.99 ± 0.312 | 62.68 ± 0.646 | 8.79 ± 0.312 |
|  | 100 | 1.17 ± 0.055 | n.d. | 6.33 ± 0.081 | 48.10 ± 0.118 | 11.91 ± 0.523 |
| S5 | 150 | 1.28 ± 0.061 | 5.25 ± 0.247 | 6.73 ± 0.623 | 61.61 ± 0.496 | 8.84 ± 0.405 |
|  | 100 | 1.16 ± 0.040 | n.d. | 6.41 ± 0.056 | 46.78 ± 0.132 | 10.57 ± 0.403 |

n.d.: not detected

**Table S5.** Genomic mutation analysis of mutant strains F2 and S3

| Strain | SNPs | InDels | C-T | Co-mutations | Synonymous mutations | Nonsynonymous mutations |
| --- | --- | --- | --- | --- | --- | --- |
| F2 | 1325 | 3860 | 353 | 981 | 155 | 106 |
| S3 | 1276 | 3909 | 344 |  | 150 | 104 |

**Supplementary Figure 1**
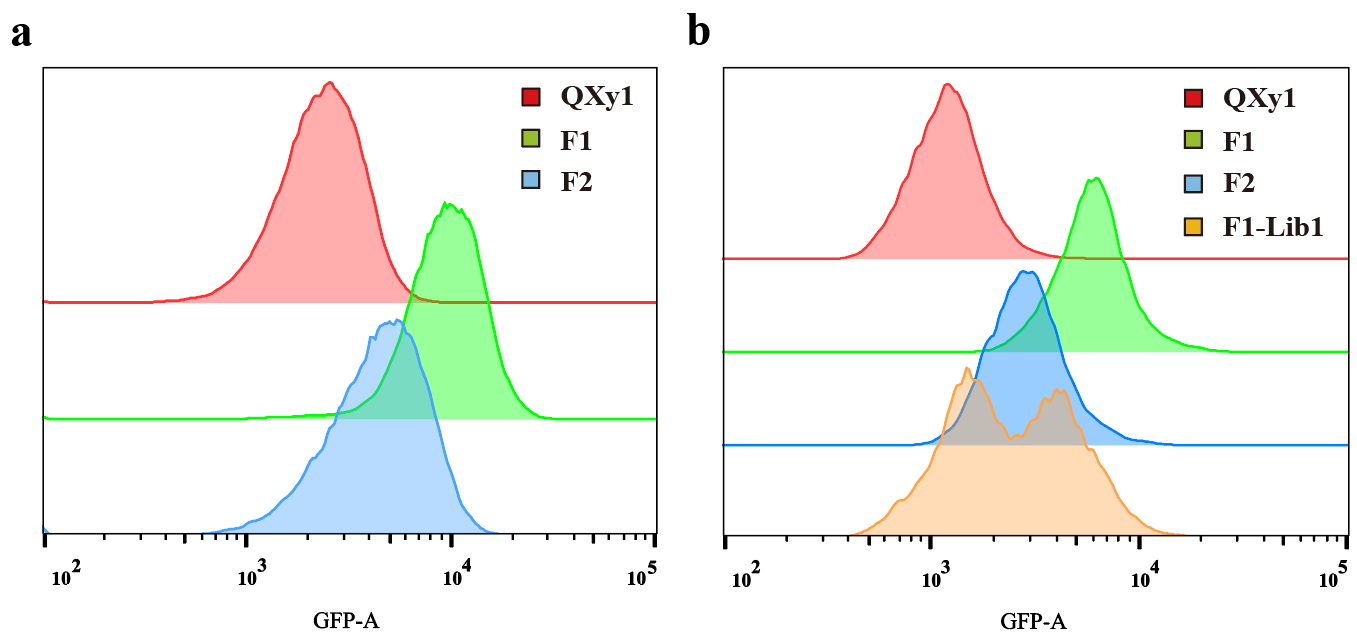


**Supplementary Figure 1.** Flow cytometry analysis of genome-wide perturbation libraries. (a) Fluorescence cytometry analysis of mutant strains F1 and F2 obtained from the first round of evolutionary screenings. (b) Flow cytometry analysis of the mutant strain F1, F2, and the second-round evolutionary library F1-Lib1. F1 (green), F2 (blue), and F1-Lib1 (orange), compared to control strains QXy1 (red).


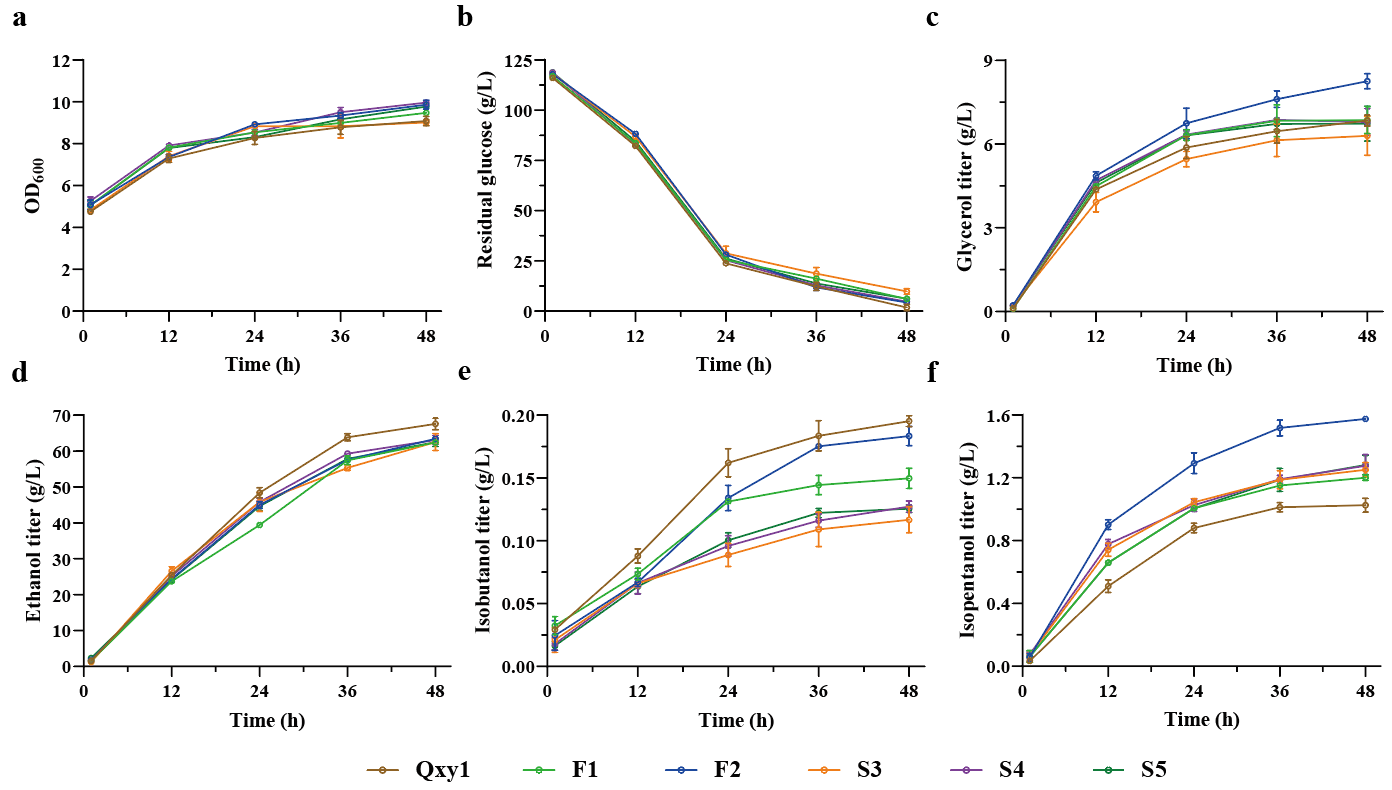
**Supplementary Figure 2**

**Supplementary Figure 2.** Continuous monitoring of the fermentation process. The fermentation performance of the control strain QXy1 (brown), mutant strains F1 (green), F2 (blue), S3 (orange), S4 (purple), and S5 (dark green) was continuously monitored at 1-hour, 12-hours, 24-hours, 36-hours, 48-hours intervals during microaerobic fermentation for (a) OD_600_, (b) Glucose titer，(c) Glycerol titer, (d) Ethanol titer, (e) Isobutanol titer, and (f) Isopentanol titer.


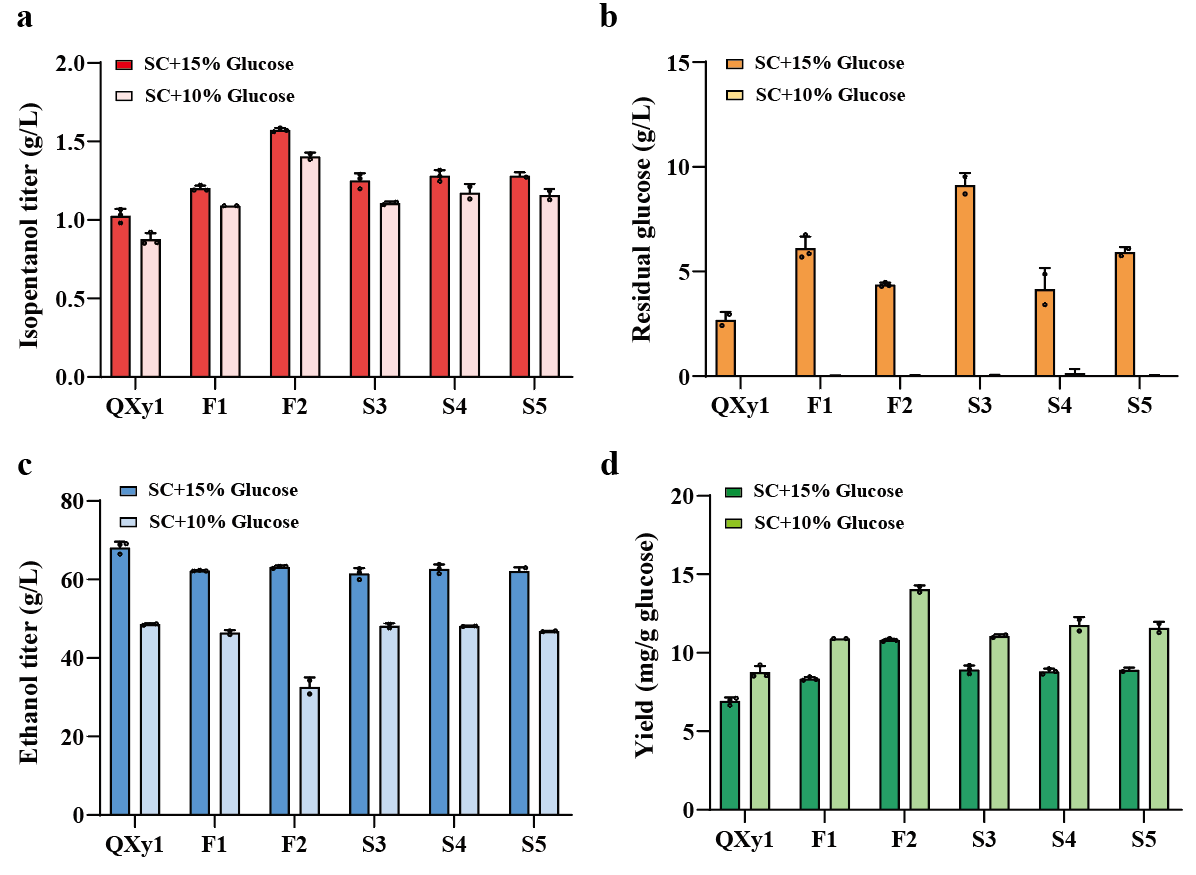
**Supplementary Figure 3**

**Supplementary Figure 3.** Optimization of glucose concentration in the fermentation medium. (a) Isopentanol titer, (b) Residual glucose, (c) Ethanol titer, and (d) Yield after 48 hours of fermentation in SC medium supplemented with 15% glucose and 10% glucose, for control QXy1 and five mutant strains respectively.


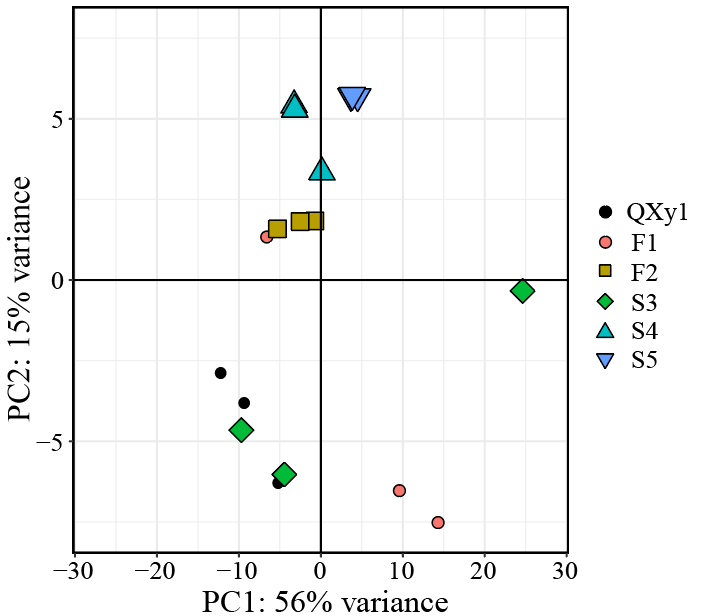
**Supplementary Figure 4**

**Supplementary Figure 4.** Principal component analysis (PCA) plot can effectively visualize differences both between and within sample groups. In our analysis, one sample from the F1 group and one from the S3 group exhibited significant outliers, likely attributable to phenotypic variations among different mono-clones. These outliers were carefully excluded to ensure the reliability and accuracy of the subsequent transcriptome analysis. Control strain QXy1 (black circles), mutant strains F1 (pink source), F2 (brown squares), S3 (green squares), S4 (dark green triangles), and S5 (blue triangles).


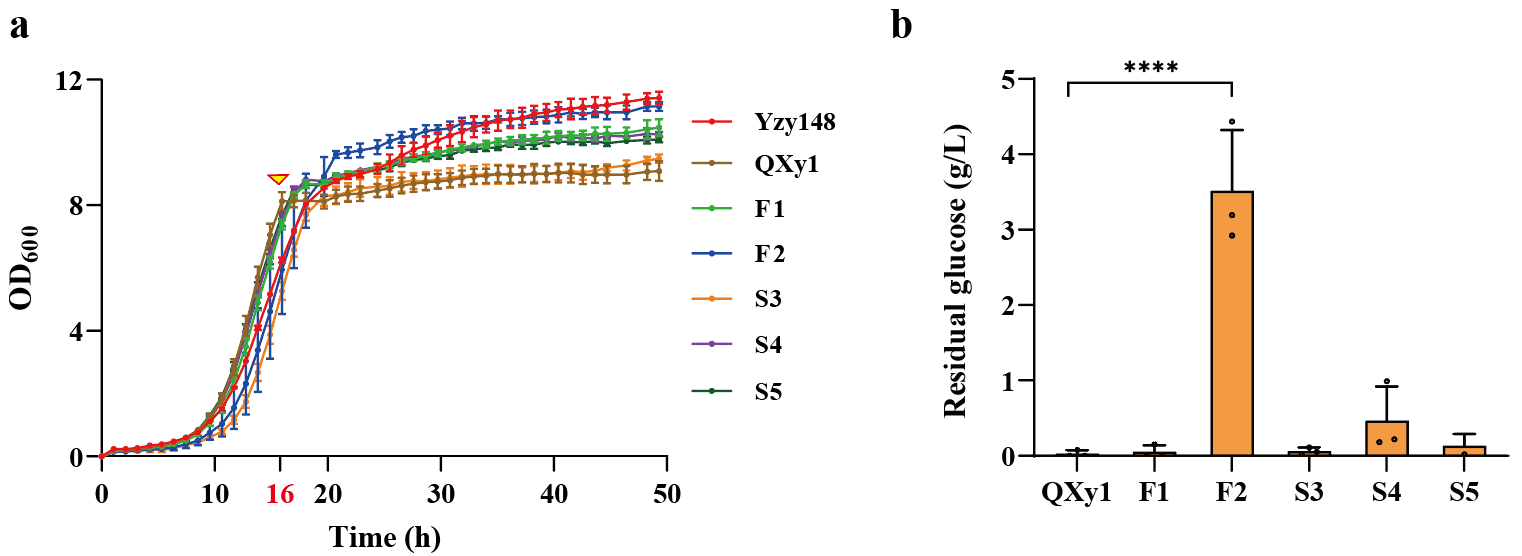
**Supplementary Figure 5**

**Supplementary Figure 5.** Glucose utilization in mutant strains. (a) Growth curves of the strains were continuously monitored under 2% glucose conditions. The strains included wild-type strain Yzy148 (red), control strain QXy1 (brown), mutant strains F1 (green), F2 (blue), S3 (orange), S4 (purple), and S5 (dark green). (b) Residual sugar concentrations of the control strain QXy1 and five mutant strains after 16 hours of incubation under 2% glucose incubation.


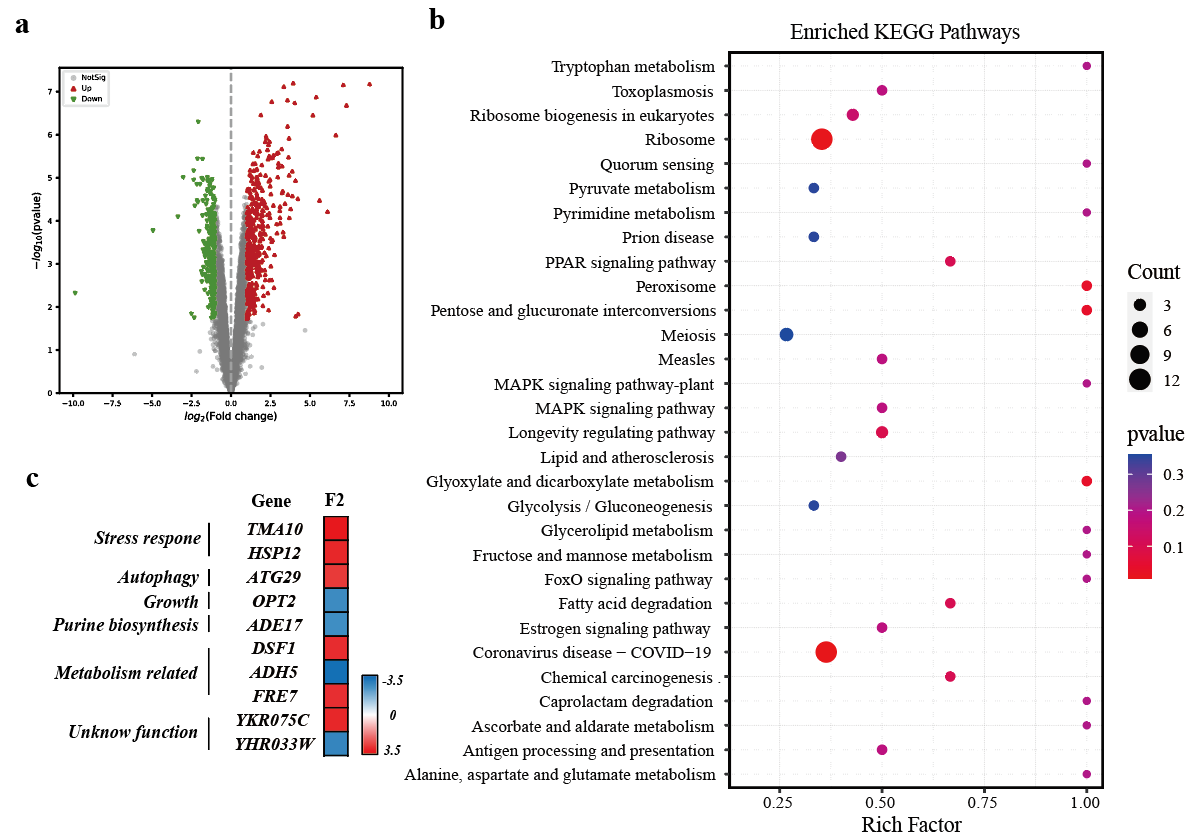
**Supplementary Figure 6**

**Supplementary Figure 6.** Transcriptome analysis of mutant strain F2. (a) Volcano plot of Differentially Expressed Genes (DEGs). Red indicates the upregulated transcription DEGs, while green indicates the downregulated transcription DEGs. (b) The KEGG pathway enrichment analysis of DEGs. (c) Functional analysis of DEGs. Red bars indicate the upregulated transcription DEGs, and blue bars indicate the downregulated transcription DEGs.

**Reference**

[1] Zhang Y, Cortez JD, Hammer SK, Carrasco-Lopez C, Garcia Echauri SA, Wiggins JB, et al. Biosensor for branched-chain amino acid metabolism in yeast and applications in isobutanol and isopentanol production. Nat Commun 2022;13(1):270. <https://doi.org/10.1038/s41467-021-27852-x>.

[2] Wang J, Zhao D, Li J, Hu M, Xin X, Price MA, et al. Helicase-AID: A novel molecular device for base editing at random genomic loci. Metabolic Engineering 2021;67:396-402. <https://doi.org/10.1016/j.ymben.2021.08.005>.
